# Draft Genome Sequence of Pseudomonas aeruginosa Strain LMG 1272, an Atypical White Line Reaction Producer

**DOI:** 10.1128/MRA.01363-19

**Published:** 2020-02-13

**Authors:** Farzaneh Salari, Fatemeh Zare-Mirakabad, Mohammad Hossein Alavi, Léa Girard, Mahya Ghafari, René De Mot, Hassan Rokni-Zadeh

**Affiliations:** a Department of Mathematics and Computer Science, Amirkabir University of Technology, Tehran, Iran; b Department of Medical Biotechnology, School of Medicine, Zanjan University of Medical Sciences, Zanjan, Iran; c Social Security Organization, Zanjan, Iran; d Department of Microbial and Molecular Systems, Faculty of Bioscience Engineering, University of Leuven, Heverlee-Leuven, Belgium; University of Arizona

## Abstract

The draft genome sequence of Pseudomonas aeruginosa LMG 1272, isolated from mushroom, is reported here. This strain triggers formation of a precipitate (“white line”) when cocultured with Pseudomonas tolaasii. However, LMG 1272 lacks the capacity to produce a cyclic lipopeptide that is typically associated with white line formation, suggesting the involvement of a different diffusible factor.

## ANNOUNCEMENT

When some Pseudomonas strains are cocultured on solid medium, a visible precipitate (“white line”) is formed between two physically separated colonies (1). This phenomenon, designated the white line reaction (WLR), presumably results from the coprecipitation of cyclic lipopeptides (CLPs) secreted by both interacting *Pseudomonas* strains, as the WLR is abolished if CLP production in one of the strains is inactivated (2). Originally, the WLR was considered specific to the interaction of the tolaasin-producing mushroom pathogen Pseudomonas tolaasii with “*Pseudomonas reactans*,” a group representing different species producing white line-inducing principle (WLIP) (3, 4). However, the WLR was also observed between producers of the tolaasin analogue sessilin and producers of orfamide (5). A WLR with P. tolaasii was even reported for the mushroom isolate NCPPB 2195, which belongs to Pseudomonas aeruginosa, an opportunistic human pathogen not known to produce CLPs (6). Here, we report the draft genome sequence of P. aeruginosa strain LMG 1272 (NCPPB 2195). Default parameters were used for all software without exception.

Strain LMG 1272, obtained from the BCCM/LMG culture collection (Belgium), was cultured in one subculture in Trypticase soy broth (TSB) or agar (TSA) medium (BD Biosciences) at 37°C. Genomic DNA was obtained from the pure broth culture using the Gentra Puregene Yeast/Bact. kit (Qiagen Benelux B.V., Venlo, The Netherlands). Genomic library preparation and sequencing were outsourced to Macrogen (Seoul, South Korea). Shotgun library preparation was performed using a TruSeq Nano DNA kit with a target insert size of 350 bp (Illumina, San Diego, CA). Paired-end sequencing (2 × 101-bp paired-end reads) was performed by an Illumina HiSeq 2000 system. In total, 2,324,619 paired-end reads were obtained. Based on FastQC version 0.11.5, all plots and reports passed the required threshold, indicating approved quality of sequencing. *De novo* assembly was performed using MaSuRCA version 2.3.2 with default settings for bacteria. Reads were mapped onto the contigs by the EIM pipeline (7) in order to generate a modified contig set. A total of 81 contigs with an *N*_50_ value of 314,105 bp (about 68-fold coverage) were generated. The final assembled length comprises 6,449,416 bp with a G+C content of 66.3%, and the longest contig size is 890,303 bp. QUAST version 5.0.2 (8) was used to compare contigs produced by the EIM pipeline and by MaSuRCA. The generated genomes were aligned by QUAST against the genome sequence of P. aeruginosa strain 19BR. The results show that EIM reduced the average numbers of mismatches and indels per 100 kb from 500.08 to 488.81 and from 12.52 to 12.14, respectively. In addition, the number of IUPAC codes was decreased from 564 to 8. Annotation of the assembled contigs using the NCBI Prokaryotic Genome Annotation Pipeline (9) identified 6,039 coding DNA sequences and 84 tRNA genes.

P. aeruginosa LMG 1272 carries homologues of pyocin genes S4 and S11 and also a homologue of F-type tailocin gene cluster (10). Genome analysis using antiSMASH 5.0 (11) to identify biosynthetic gene clusters of secondary metabolites revealed the capacity to produce pyoluteorin (12), pyochelin (13), 2-amino-4-methoxy-*trans*-3-butenoic acid (14), and azabicyclene (15). The lack of characteristic CLP biosynthetic genes (16) indicates that a different diffusible factor is involved in the WLR phenotype.

### Data availability.

This whole-genome shotgun project has been deposited in DDBJ/ENA/GenBank under accession number VIYL00000000. The version described in this paper is version VIYL01000000. The raw sequencing data are available from the Sequence Read Archive (SRA) under accession number PRJNA552675.
